# Laser Speckle Imaging of Sensitized Acupoints

**DOI:** 10.1155/2018/7308767

**Published:** 2018-07-17

**Authors:** Ning Ding, Jing Jiang, Xiaoxiao Liu, Yiyuan Xu, Jiatong Hu, Zhigang Li

**Affiliations:** Beijing University of Chinese Medicine, Beijing 100029, China

## Abstract

Acupoints microcirculatory dynamics vary depending on the body's health status. However, the functional changes observed during acupoint sensitization, that is, the disease-induced change from a “silenced” to an “activated” status, remain elusive. In this study, the microcirculatory changes at acupoints during sensitization were characterized. Thirty SD rats were randomly divided into five groups: normal control group (N), sham osteoarthritis group (S), light osteoarthritis group (A), mild osteoarthritis group (B), and heavy osteoarthritis group (C). The obtained results showed that the blood perfusion levels at the acupoints Yanglingquan (GB34), Zusanli (ST36), and Heding (EX-LE2) in groups A, B, and C were higher than those in groups N and S on days 14, 21, and 28 (p < 0.01 or p < 0.05). A significant difference in the blood perfusion was also observed at the acupoint Weizhong (BL40) in groups B and C on days 21 and 28 (p < 0.01). In addition, remarkable differences in the level of blood perfusion at the GB34, ST36, and EX-LE2 acupoints were observed on day 28 (p < 0.01 or p < 0.05) among groups A, B, and C. No marked differences in blood perfusion levels were observed at the nonacupoint site among all groups. In conclusion, acupoint sensitization is associated with an increase in the level of local blood perfusion at specific acupoints, and this increase is positively correlated with the severity of the disease. The functional changes in microcirculation at acupoints during sensitization reflect the different physiological and pathological conditions imposed by the disease.

## 1. Introduction

As an important functional unit of the circulatory system, the microcirculation plays a pivotal role in the nutrient supply, maintenance of active cell metabolism, and the regulation of homeostasis. Microcirculation is a significant target for acupuncture-based therapies. Many studies focused on the microcirculation dynamics in order to unravel the underlying mechanisms of acupuncture. It has been suggested that acupuncture can not only regulate the blood flow through the microcirculatory system in acupoints [1, 2] but also affect the blood perfusion level across the entire affected meridian [3]. Additionally, the modulation of blood perfusion rates in effector organs has been reported as one of the important mechanisms of acupuncture-based therapies [4, 5].

It is worthwhile to point that, as compared with the surrounding tissue, acupoint has special microcirculatory features [6]. An acupoint can be regarded as a dynamic portal in time and body location, which greatly varies according to different physiological and pathological conditions [7, 8]. However, current studies mainly focus on the microcirculatory effect of acupuncture on acupoints or on effector organs. The physiologically and pathologically induced microcirculatory changes that occur at acupoints before acupuncture intervention have not been characterized. Acupoint sensitization refers to the dynamic switch from a “silenced” status (under normal conditions) to an “activated” status (under unfavorable physiological and pathological conditions), that is, the response of acupoints to disease [8, 9]. There is ample evidence to support the fact that the acupoint sensitization markedly affects the area of an acupoint's receptive field, its level of responsiveness to treatment, and, ultimately, the effectiveness of an acupuncture-based therapy [10, 11]. Therefore, the level of acupoint sensitization determines its ability to sense and respond to external stimuli as well as its ability to contribute to the restoration of body functions. Additionally, sensitized acupoints are considered as disease markers on the body surface and therefore are of clinical value for both diagnosis and treatment [12, 13].

The underlying mechanisms of acupoint sensitization have been studied to some extent. Studies have shown that acupoint sensitization is dependent on the neuronal response at the spinal and medulla levels. Both the wide dynamic range (WDR) neurons in the dorsal horn and the subnucleus reticularis dorsalis (SRD) neurons in the caudal portion of the medulla neurons play an important role in acupoint sensitization during visceral nociception [8–10]. Despite its technical limitations, the imaging analysis provides a direct visual characterization of the dynamic changes occurring during acupoint sensitization. In addition to a full visual characterization of the acupoint sensitization dynamics under a particular disease, the imaging analysis also allows for the quantification of the level of response at acupoints, which could be used as an indicator of the severity of a disease. Therefore, the imaging analysis of acupoint sensitization might have wide application in the clinical practices that include an improved diagnosis and treatment. Besides, a better visual characterization of the microcirculation dynamics at local acupoints adds to the current understanding of acupuncture practices and associated mechanisms.

In the present study, we investigated the microcirculatory changes in the acupoints Yanglingquan (GB34), Zusanli (ST36), Heding (EX-LE2), and Weizhong (BL40) and the nonacupoint site during sensitization to monoiodoacetate- (MIA-) induced knee osteoarthritis at different degrees of disease severity. Real-time laser speckle imaging was used to measure the level of blood perfusion at local acupoints* in vivo* at various time intervals. For monitoring the severity of disease, serum tumor necrosis factor-*α* (TNF-*α*) and the cartilage oligomeric matrix protein (COMP) were assessed by ELISA.

## 2. Materials and Methods

### 2.1. Experimental Animals

Sprague-Dawley (SD) rats were purchased from the SPF (Beijing) Biotechnology Co., Ltd. (Animal Lot: SCXK(Jing)2016-0002). SD rats weighed 150.0±10.0 g. The animals were housed in a fenced facility in the Experimental Animal Center of Beijing University of Chinese Medicine at a controlled temperature (24±2°C) and under a 12-hour dark/light cycle, with sterile drinking water and a standard pellet diet available ad libitum. All rats were acclimatized to the environment for 7 days prior to experimentation, and all experimental procedures complied with the guidelines of the “Principles of Laboratory Animal Care” formulated by the National Institutes of Health and the legislation of the People's Republic of China for the use and care of laboratory animals. The experimental protocols were approved by the Medicine and Animal Ethics Committee of Beijing University of Chinese Medicine. Efforts were made to minimize the number of animal uses and the suffering of the experimental animals.

### 2.2. Animal Grouping and Intervention

Thirty SD male rats were divided into five groups randomly (*n* = 6 per group): the normal control group (N), the sham osteoarthritis group (S), the light osteoarthritis group (A), the mild osteoarthritis group (B), and the heavy osteoarthritis group (C).

All interventions were carried out after laser speckle imaging on day 0. According to Guingamp [14], MIA (Sigma, St. Louis, USA) was dissolved in 50 *μ*l of sterile saline water. MIA solution was injected into the right knee joint through the infrapatellar ligament. The rats in groups A, B, and C were injected with 0.3, 1, and 3 mg of MIA, respectively, and the rats in group S were injected with 50 *μ*l of saline solution. The various doses of MIA were in accordance with Pomonis [15]. No intervention was carried out in group N.

### 2.3. ELISA Assay

On days 0, 7, 14, 21, and 28, all rats were intraperitoneally anesthetized with 20% urethane. A blood sample was taken from the medial canthus and then centrifuged at 3000 rpm, at 4°C, for 10 min for the separation of serum. The levels of TNF-*α* and COMP were measured in the serum with an ELISA kit (Oubei Biotechnology Co., Ltd, Beijing). First, 50 *μ*l of an antibody operating solution was added to each sample at room temperature for 120 minutes. Subsequently, 100 *μ*l of a horseradish peroxidase-labeled secondary antibody solution was added to each sample at room temperature, in the dark, for 30 minutes. Complete plate washing was performed after four attempts. A 100 *μ*l chromogenic substrate operating solution was added to each plate at room temperature under dark condition for 30 minutes. Plate washing was performed after four completed attempts. Then a total of 100 *μ*L of stop solution was added to each sample. The absorbance values were measured (450 nm) using a microplate reader (Thermo Corporation, USA) within 30 minutes of sample preparation. A standard curve was produced in Excel by plotting known concentrations against the absorbance values of the standard solutions prepared from purified protein supplied with the ELISA kit. The unknown TNF-*α* and COMP protein concentrations were estimated using the equation associated with the calibration curve.

### 2.4. Laser Speckle Imaging

A laser speckle imaging system (PeriCam PSI, Perimed) was used to monitor the rate of blood perfusion at GB34, ST36, EX-LE2, and BL40 and the nonacupoint site on days 0, 7, 14, 21, and 28. The room temperature was controlled at 25 ± 1°C, whereas the relative humidity was 55 ± 10 %, and wind speed was < 1 m/s. The laser speckle system that was set at a wavelength of 785 nm offered a video-frame rate visualization of blood flow at 21 frames per second with a resolution of 1388 × 1038 pixels. The working distance between the scan head and the rat lower limbs varied between 15 and 20 cm. The monitoring area of the ventral and dorsal side of lower limb was 5 × 7 cm, where each area was monitored for longer than 1 min. The rats were positioned below the laser speckle probe during imaging and the movement in the lower limbs was restrained after anesthesia. Body hair in lower limbs was removed 24 h before the induction of anesthesia. GB34, ST36, EX-LE2, and BL40 and the nonacupoint site were selected according to previous studies [16, 17] and identified by the use of a pointer in the beginning of the laser speckle imaging scanning for location accuracy purposes. After the blood flow images were recorded, the locations of GB34, ST36, EX-LE2, and BL40 and the nonacupoint site were defined on the images. The blood perfusion level in each acupoint, at a 1.5 × 1.5 mm scale, was analyzed with signal processing software (PIMSoft 2.0, Perimed AB, Sweden). To measure the level of sensitization, the following equation was used:

(1) $$\begin{array}{c} A=\frac{a-b}{b}\times 100\% \end{array}$$

where “A” is the level of sensitization, “a” is the mean of the blood perfusion level of a group (S, A, B, or C), and “b” is the mean of the blood perfusion level of group N at the same time point.

### 2.5. Statistical Analysis

The statistical analysis was performed using the SPSS software, version 17.0 (SPSS, Inc., Chicago, IL, USA), and the data were expressed as the mean ± standard deviation. A one-way ANOVA was used after the normal distribution and homogeneity of variance were confirmed. For the nonnormally distributed data or for data with heterogeneous variance, a nonparametric test was used. The LSD method was applied for pairwise comparisons. Statistical significance was set to p < 0.05 and high statistical significance was set to p < 0.01.

## 3. Results

### 3.1. The Serum Contents of TNF-*α* and COMP from Day 0 to Day 28

**Figure 1:**
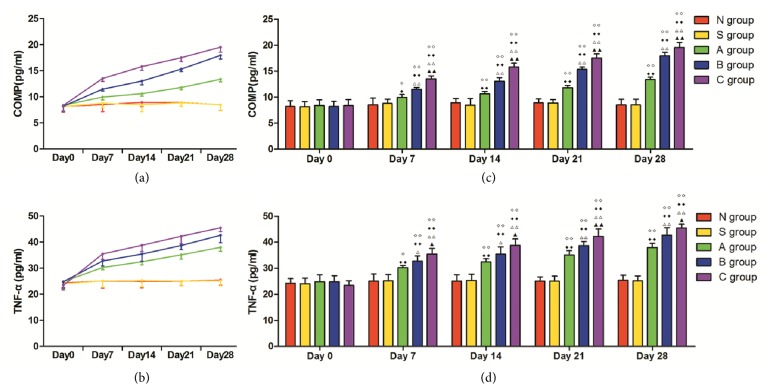
Serum levels of TNF-*α* and COMP in each group throughout the experimental period (*n* = 6, mean ± SD). ((a) and (b)) Trend in the serum level of COMP and TNF-*α*. ((c) and (d)) Histogram representing the serum content of COMP and TNF-*α*. ^*◇◇*^P < 0.01 and ^*◇*^P < 0.05 compared with group N. ^◆◆^P < 0.01 and ^◆^P < 0.05 compared with group S. ^△△^P < 0.01 and ^△^P < 0.05 compared with group A. ^▲▲^P < 0.01 and ^▲^P < 0.05 compared with group B.

The contents of TNF-*α* and COMP in each group are presented in Figure 1. The contents of TNF-*α* and COMP in groups A, B, and C increased gradually from day 0 to day 28. No changes were observed in groups N and S throughout the experimental period. The serum contents of TNF-*α* and COMP in groups A, B, and C were statistically higher than those in groups N and S from day 7 to day 28 (p < 0.01 or p < 0.05). The levels of TNF-*α* and COMP in the serum of group A were significantly lower than those of groups B and C at all time points (p < 0.01 or p < 0.05), whereas group B was lower than group C (p < 0.01 or p < 0.05).

### 3.2. Blood Perfusion at GB34, ST36, EX-LE2, and BL40 and the Nonacupoint Site from Day 0 to Day 28

**Figure 2:**
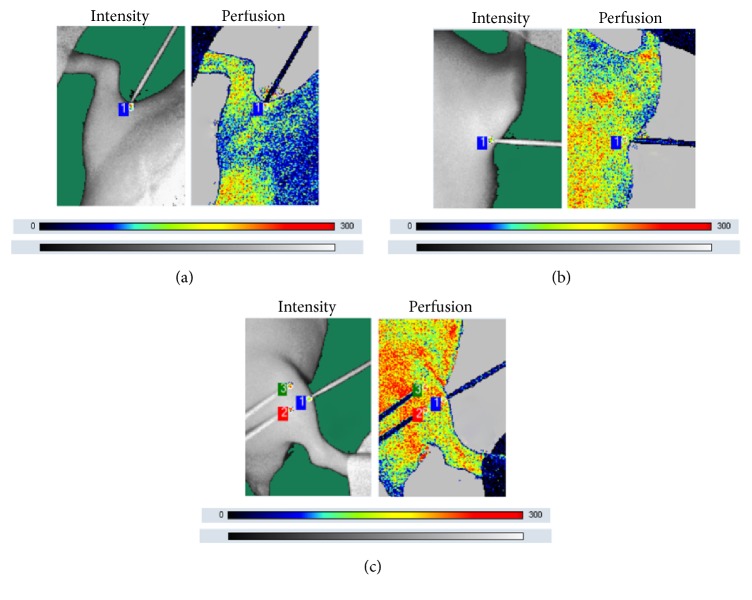
Laser speckle imaging of GB34, ST36, EX-LE2, and BL40 and the nonacupoint site. (a) Laser speckle imaging of BL40 (1). BL40 is located at the back of the knee, on the popliteal crease, in a depression midway between the tendons of biceps femoris and semitendinosus. (b) Laser speckle imaging of EX-LE2 (1). EX-LE2 is at the midpoint of the superior border of the patella. (c) Laser speckle imaging of ST36 (1) and GB34 (3) and the nonacupoint site (2). ST36 is located in the anterior tibial muscle and 5 mm lateral and distal to the anterior tubercle of the tibia. GB34 is at the depression below the capitulum fibulae posterolateral to the knee joint. The nonacupoint site is located at 5 mm next to ST36 outside.

**Figure 3:**
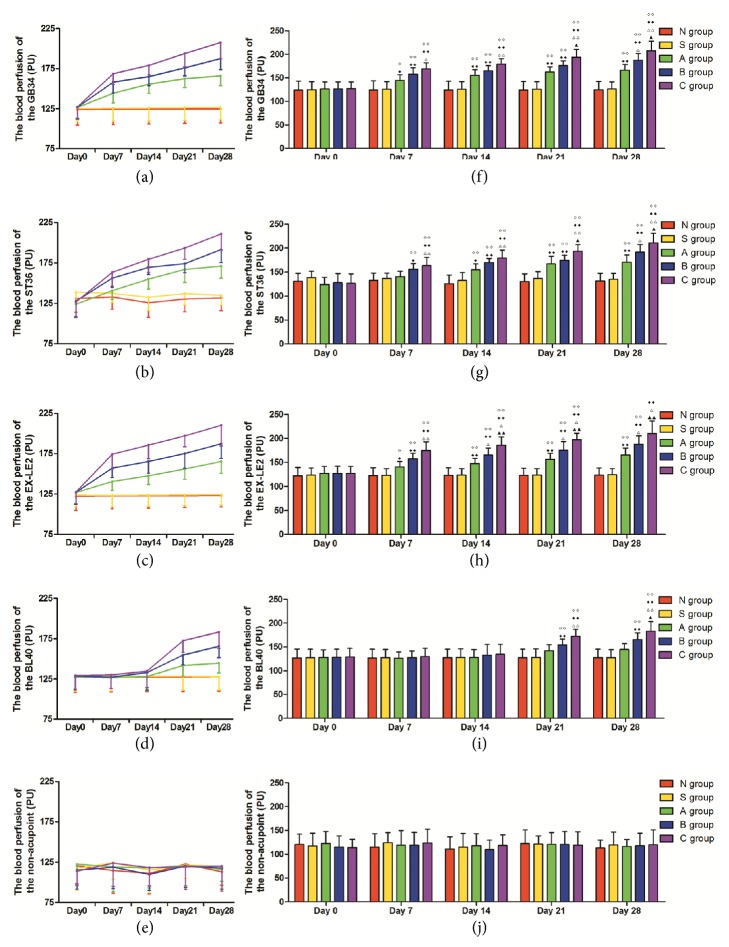
Comparison of flood perfusion rates at GB34, ST36, EX-LE2, and BL40 and the nonacupoint site in all groups throughout the experimental period (*n* = 6, mean ± SD). (a)–(e) Trend of change in the blood perfusion level at GB34, ST36, EX-LE2, and BL40 and the nonacupoint site. (f)–(j) Histogram representing changes in the blood perfusion level at GB34, ST36, EX-LE2, and BL40 and the nonacupoint site. ^*◇◇*^P < 0.01 and ^*◇*^P < 0.05 compared with group N. ^◆◆^P < 0.01 and ^◆^P < 0.05 compared with group S. ^△△^P < 0.01 and ^△^P < 0.05 compared with group A. ^▲▲^P < 0.01 and ^▲^P < 0.05 compared with group B.

Blood perfusion results at GB34, ST36, EX-LE2, and BL40 and the nonacupoint site in each group are presented in Figures 2 and 3. No significant changes were observed in the blood perfusion levels at GB34, ST36, EX-LE2, and BL40 among all groups on day 0. The blood perfusion level of GB34, ST36, and EX-LE2 in groups A, B, and C increased gradually from day 0 to day 28; however, such changes were observed at BL40 from day 21 to day 28. There were no remarkable changes in blood perfusion levels at GB34, ST36, EX-LE2, and BL40 between groups N and S throughout the experimental period. No significant changes in the blood perfusion levels at the nonacupoint site were observed from day 0 to day 28 in any of the experimental groups. The blood perfusion levels at GB34, ST36, and EX-LE2 in groups A, B, and C were higher than those in groups N and S from day 7 to day 28 (p < 0.01 or p < 0.05) except ST36 in group A on day 7. In BL40, blood perfusion levels of groups B and C increased markedly as compared to groups N and S on days 21 and 28 (p < 0.01). The blood perfusion level at GB34 and ST36 in group C was higher than that in group A (p < 0.01 or p < 0.05) from day 7 to day 28 and higher than that of group B on days 21 and 28 (p < 0.05). Remarkable differences at the GB34 and ST36 were observed on day 28 between groups A and B (p < 0.05). The blood perfusion level of EX-LE2 in group C was higher than that of group A on day 7 (p < 0.01) and higher than those of groups A and B on days 14, 21, and 28 (p < 0.01 or p < 0.05). Significant differences in the blood perfusion level at EX-LE2 were observed between groups A and B on days 14, 21, and 28 (p < 0.05). The blood perfusion level of BL40 in group C was higher than that of group A on day 21 (p < 0.01) and higher than those in groups A and B on day 28 (p < 0.01 or p < 0.05).

### 3.3. The Level of Sensitization at GB34, ST36, EX-LE2, and BL40 and the Nonacupoint Site from Day 0 to Day 28

**Figure 4:**
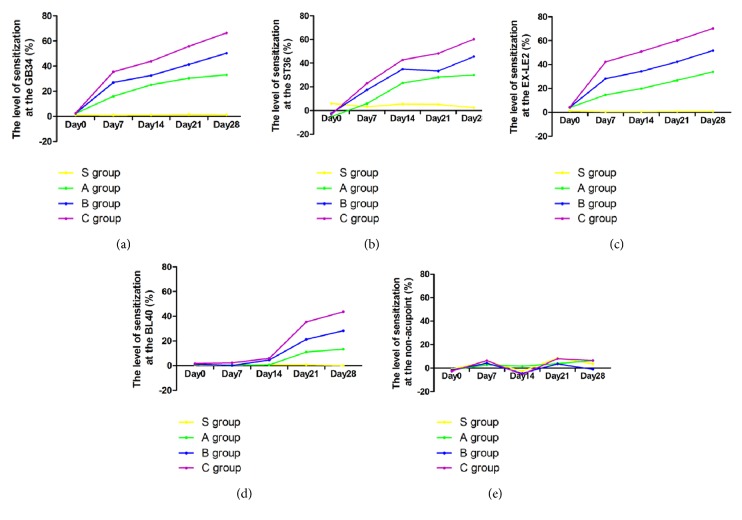
The level of sensitization at GB34, ST36, EX-LE2, and BL40 and the nonacupoint site in all the groups throughout the experimental period. (a)–(e) Trend of change in the level of sensitization at GB34, ST36, EX-LE2, and BL40 and the nonacupoint site.

The levels of sensitization at GB34, ST36, EX-LE2, and BL40 and the nonacupoint site in each group are presented in Figure 4. On day 0, the level of sensitization at the above acupoints and the nonacupoint site in each group varied between -5.22% (ST36 of group C) and 5.99% (ST36 of group S). From day 7 to day 28, no obvious changes at GB34, ST36, EX-LE2, and BL40 and the nonacupoint site in group S were observed, in which the level of sensitization varied between -2.59% (the nonacupoint site on day 14) and 8.53% (the nonacupoint site on day 21). Also, there were no clear changes at the nonacupoint site in groups A, B, and C, in which the level of sensitization varied between -5.48% (group C on day 14) and 8.01% (group C on day 21). The level of sensitization at the above acupoints in groups A, B, and C increased with time. Among the acupoints that showed significant changes in the blood perfusion level, the level of sensitization varied between 14.65% (EX-LE2 of group A on day 7) and 70.23% (EX-LE2 of group C on day 28).

## 4. Discussion

Acupuncture has been widely used as an effective therapy to treat knee osteoarthritis all over the world. A growing body of evidence has demonstrated that acupuncture can alleviate knee osteoarthritis-related pain and improve knee function [18, 19] with few adverse side effects [20, 21] and it is often acting in a synergic manner with medications [22]. According to Traditional Chinese Medicine, acupoints have a dual role as disease severity indicators and as regulators of the body function [9]. Accordingly, the acupoints are important “replication” sites specific to a disease. There are a large number of clinical studies focused on treating knee osteoarthritis by acupuncture methods for which specific acupoints have been extensively selected. In particular, the treatment of knee osteoarthritis with acupuncture focuses mainly on the acupoints around the knee, specifically GB34, ST36, EX-LE2, and BL40 [23–26]. Therefore, these acupoints, depending on how often they are used in the acupuncture treatment of knee osteoarthritis [27], were selected as the target acupoints in our study. In addition, a nonacupoint site control was chosen in order to take into account the influence of an increase in the inflammatory mediators in the blood after exposure to disease.

As an extracellular noncollagen cartilage matrix protein, COMP has a role in the dedifferentiation of articular chondrocytes. Recent studies showed that serum levels of COMP are elevated in patients with knee osteoarthritis. Moreover, the levels of COMP increase with the severity of the disease and thus it is regarded as a diagnostic and prognostic biomarker in knee osteoarthritis [28, 29]. TNF-*α* is an important mediator of cartilage matrix degradation and tissues degeneration in the inflammatory joints disorders. Elevated levels of serum TNF-*α* are observed in patients with knee osteoarthritis, which, similar to COMP, also increase with the severity of disease [30]. The ELISA results in this study showed no remarkable differences in the serum level of COMP and TNF-*α* of all the groups on day 0, which indicates that, at the beginning of the experiment, the health status of the knees of rats was equally good among the experimental groups. Also, there were no significant differences in the levels of COMP and TNF-*α* between groups N and S, suggesting that group S could be used as an effective control to groups A, B, and C as it allowed for the exclusion of possible inflammatory response brought by the injection itself. The serum levels of COMP and TNF-*α* in groups A, B, and C were consistently and statistically higher than those of groups N and S from days 7 to 28 (p < 0.01 or p < 0.05). These results strongly suggested that the rats in these groups developed inflammatory responses and suffered cartilage damage as seen in knee osteoarthritis. In addition, the levels of COMP and TNF-*α* varied significantly among groups A, B, and C (p < 0.01 or P < 0.05), which suggested that the injection with increasing amounts of MIA caused knee osteoarthritis of increasing severity (light, mild, and heavy osteoarthritis). These results confirmed that the level of induced osteoarthritis was positively correlated to the injected amount of MIA and is consistent with the previous studies [15]. Altogether, the data demonstrated that we successfully created a reliable experimental knee osteoarthritis model for the study of acupoint sensitization.

Results of the laser speckle imaging analysis showed that the blood perfusion levels at all the tested acupoints, GB34, ST36, EX-LE2, and BL40, were significantly higher in groups A, B, and C than in groups N and S at the corresponding time points (p < 0.01 or p < 0.05). These results indicated that the microcirculatory function, at specific acupoints, is affected by the disease and could therefore be used as an indicator of the physiological and pathological status of the body. The laser speckle imaging analysis undertaken in this study allowed, for the first time, the* in vivo*, real-time visualization of the dynamic changes, which occur during sensitization at the acupoint as a response to disease. In particular, these results confirmed that acupoint sensitization is associated with an increase in the local blood perfusion level, and, therefore, an increase in the microcirculatory function should be considered as the physiological mechanism underlying acupoint sensitization. Our results suggested that the blood perfusion levels during sensitization varied between GB34, ST36, EX-LE2, and BL40, with GB34, ST36, and EX-LE2 responding faster than BL40, which is basically in agreement with the frequency, and these acupoints are selected for the treatment of knee osteoarthritis in the current acupuncture practices [27]. Meanwhile, no significant difference was observed in the blood perfusion level at the nonacupoint site, which strongly suggested that the changes in the blood perfusion level are limited to sensitized acupoints. These results indicated that acupoint sensitization is specific, both in response intensity and in the rate of development. Such specificity may provide important diagnostic clues during clinical practices. Also, the level of correlation between the blood perfusion level and the severity of the disease differed among the acupoints. Strong positive correlations between the level of blood perfusion and disease were found out at EX-LE2, GB34, and ST36 in a decreasing order. BL40 did not show such a strong correlation between the level of sensitization and disease severity as the other acupoints. These results demonstrated that the changes in the microcirculatory function during acupoint sensitization can reflect the severity of a disease to a certain extent and hold great promise in improving the capability of diagnosis in clinical practices of acupuncture. Furthermore, the blood perfusion dynamics within the same acupoint also changed throughout the experimental period in all levels of disease severity. The dynamic characteristics of acupoints sensitization were expressed in the changes in the microcirculatory function of specific acupoints during sensitization, which varies with different physiological and pathological conditions. The dynamic nature of acupoints sensitization might further enhance their value as sensitive indicators of disease as their level of sensitization closely correlates to the body state.

In addition, our results showed that, among the acupoints that had significant changes in the blood perfusion level, the level of sensitization varied between 14.65% (EX-LE2 of group A on day 7) and 70.23% (EX-LE2 of group C on day 28). This rate of change in sensitization status suggests that a 15% increase in the blood perfusion level might be used as the minimum level of blood profusion increase above which an acupoint is considered as sensitized.

As for the underlying mechanism regulating the microcirculation during acupoint sensitization, the compound structures of MCs, nervous fibers, and vessels in the acupoint have been observed, and the interrelations and interactions between the circulatory system, immune system, and nervous system at the acupoint are considered as histological characteristics of the acupoint and the morphological basis in response to acupuncture [31, 32]. Specifically, we deduce that nociceptive stimulus from the joint tissue conducts to posterior horn of spinal cord through dorsal root ganglion and then antidromically conducts to peripheral nerve through dorsal root reflex and axon reflex. It can promote the release of inflammatory substances from the nerve endings at the acupoints such as substance P, calcitonin gene-related peptide. On one hand, the release of such substances at the acupoints has a regulatory effect on the blood flow [33, 34]. On the other hand, MCs are activated [35]. A series of neurotransmitters such as 5-hydroxytryptamine (5-HT) [36] and histamine (HA) [37], released during degranulation of mast cells (MCs), are also vasoactive. Therefore, the changes in local microcirculation during acupoint sensitization seem to be a result of complex interactions between the circulatory system, immune system, and nervous system, further reinforcing the notion that acupoint sensitization is multileveled and multifaceted, though this is not yet well understood.

The laser speckle imaging analysis used in this study is currently the main visualization technique of blood flow. It has the advantages of allowing a wide field-of-view and of having high spatial and temporal resolution. However, it is incapable of producing a depth-resolved image of microvessels and is insensitive to motion artifacts [38, 39]. Limited by these methodological disadvantages, this study cannot add to the current understanding of structural alterations associated with microcirculatory changes at acupoints. Nevertheless, this study allowed for the visualization of changes in the blood perfusion levels during acupoint sensitization as a response to disease (knee osteoarthritis), which might help elucidate the effectiveness of acupuncture-based therapies. The continuous improvement of imaging technologies might allow for* in vivo*, real-time analysis of microcirculatory changes occurring during acupoint sensitization in the future.

## 5. Conclusions

Based on a knee osteoarthritis model induced by MIA in rats, this study allowed for the in vivo, real-time visualization of the microcirculatory dynamics of acupoint sensitization by laser speckle imaging analysis. In particular, acupoint sensitization is associated with an increase in the local blood perfusion level; an increase above 15% can be considered as the minimum increment associated with acupoint sensitization. Besides, we propose that acupoint sensitization to a particular disease is location-specific and will vary in response intensity and the rate of development. This specificity increases the potential use for sensitized acupoints in the clinical prognosis and therapy. Additionally, the microcirculatory changes during acupoint sensitization positively correlate with severity of disease, which also points out the potential diagnostic value of sensitized acupoints in the clinical practices.

## Data Availability

The data used to support the findings of this study are included within the article.
